# The association between social integration and neighborhood dissatisfaction and unsafety: a cross-sectional survey study among social housing residents in Denmark

**DOI:** 10.1186/s13690-022-00945-9

**Published:** 2022-08-12

**Authors:** Abirami Srivarathan, Maria Kristiansen, Terese Sara Høj Jørgensen, Rikke Lund

**Affiliations:** 1 Section for Health Services Research, Department of Public Health, Faculty of Health and Medical Sciences, University of Copenhagen, Øster Farimagsgade 5, 1014 Copenhagen K, Denmark; 2 Center for Healthy Aging, University of Copenhagen, Blegdamsvej 3B, 2200 Copenhagen N, Denmark; 3 Section of Social Medicine, Department of Public Health, Faculty of Health and Medical Sciences, University of Copenhagen, Øster Farimagsgade 5, 1014 Copenhagen K, Denmark

**Keywords:** Social Determinants of Health, Social Integration, Neighborhood Environment, Social Housing, Disadvantaged Neighborhoods, Health Policy

## Abstract

**Background:**

Social integration and perceived neighborhood environment are recognized as important social determinants of health. However, little is known about the association between social integration and perceived neighborhood environment among underrepresented population groups, such as residents in disadvantaged neighborhoods, in public health research. The aim of this study is to: 1) Describe the levels of social integration and 2) Investigate the association between social integration and neighborhood dissatisfaction and unsafety among middle-aged and older social housing residents.

**Methods:**

A multilingual face-to-face interviewer-administrated survey questionnaire was conducted among 206 residents aged 45 years and above (response rate: 34.1%) of various nationalities in disadvantaged socioeconomic positions in a social housing area in Denmark. The assessment of social integration was based on cohabitation status, frequency of face-to-face and non-face-to-face interaction with social relations and participation in local association activities. Neighborhood dissatisfaction measured the level of dissatisfaction with the neighborhood, and neighborhood unsafety assessed the level of unsafety being outdoors in the neighborhood. Descriptive statistics were conducted to illustrate respondent characteristics and the distribution of social integration among the study population. Logistic regression models were applied to analyze associations between social integration and neighborhood dissatisfaction and unsafety, adjusted for age, sex, country of origin, educational attainment and employment status.

**Results:**

In total, 23.8% of the respondents reported low levels of social integration. A medium level of social integration was associated with higher odds of neighborhood dissatisfaction (OR: 2.36; 95% CI: 1.04–5.38) compared to the highest level of integration. A low frequency of face-to-face interaction was associated with higher odds of neighborhood dissatisfaction (OR: 2.65; 95% CI: 1.16–6.06) and neighborhood unsafety (OR: 2.41; 95% CI: 1.04–5.57) compared to the highest frequency of face-to-face interaction.

**Conclusions:**

Almost one-fourth of respondents reported low levels of social integration. A medium level of social integration was associated with neighborhood dissatisfaction. A low frequency of face-to-face interaction was associated with neighborhood dissatisfaction and unsafety. The results suggest that targeted health promotion interventions designed to foster face-to-face interaction, hold potential to reduce neighborhood dissatisfaction and unsafety among residents in disadvantaged neighborhoods.

**Supplementary Information:**

The online version contains supplementary material available at 10.1186/s13690-022-00945-9.

## Background 

Social integration is widely recognized as an important social determinant of health, and successful social integration has significant impact on the health and well-being of individuals [1–6]. Social integration, as conceptualized in this article, refers to the network of social relations that surrounds an individual and indicates the level of connectedness to other people [1, 6–9]. Marital status, interaction with family and friends and participation in local association activities are used as characteristics of social integration [1, 6, 7, 9, 10].

In recent decades, there has been an increasing public health focus on the significance of social integration in terms of neighborhood relationships and participation in local association activities for maintaining and improving the quality of life [2, 3, 5, 11–14]. This has been motivated by the recognition that social structures affect health and well-being [1–3, 5, 8, 15]. For residents in disadvantaged neighborhoods, where socioeconomic deprivation, housing instability and crime are comparatively more common, social relations are important for assisting people in getting by and meeting basic needs in everyday life [16–21]. In light of the material hardship and the limited economic opportunity that often challenge residents in disadvantaged neighborhoods, these residents are found to rely more on neighbors and fellow residents for material and psychosocial support compared to other population groups [16–18, 21].

A growing body of scientific literature suggests that subjective evaluations of the neighborhood environment such as perceived neighborhood dissatisfaction and unsafety are associated with health and well-being [8, 11–14, 22]. At the individual level, residents who express dissatisfaction with their neighborhood report lower quality of life and poorer mental health [11–14, 23, 24]. Residents who perceive their neighborhood as unsafe more often report feelings of psychological distress and poor self-rated health [8, 22]. Factors such as housing instability, caused by the relocation of residents and the demolition of housing, together with social disconnectedness and crime are associated with neighborhood dissatisfaction and unsafety [8, 15, 25].

Particularly in disadvantaged neighborhoods, it may be relevant to investigate the importance of social integration for neighborhood dissatisfaction and unsafety, since these outcomes have been found to be more prevalent among residents in disadvantaged neighborhoods [8, 13, 22]. Additionally, previous research has shown divergent levels of social integration in disadvantaged neighborhoods [2, 3, 16–21]. A number of studies conducted in disadvantaged neighborhoods in Denmark identified low levels of social integration among the residents [2, 3], while studies from the United States found higher levels of social integration in disadvantaged neighborhoods [16–19, 21]. These inconclusive perspectives highlight the need for studies investigating the levels of social integration among residents in disadvantaged neighborhoods in public health research. Knowledge about social integration can provide insight into the importance of social relations among residents in disadvantaged neighborhoods and the impact this has on their perceptions of the neighborhood environment. Such knowledge is needed to develop targeted health promotion interventions that enhance social integration and increase neighborhood satisfaction and feelings of safety among residents in disadvantaged neighborhoods.

This study is placed in the intersection of the following social determinants of health: social integration and neighborhood environment. An intersection that is rather unexplored in public health research [8, 22, 26]. There is a paucity of empirical studies investigating the importance of social integration for perceptions of the neighborhood environment among underrepresented population groups such as residents in disadvantaged neighborhoods. Such underrepresentation might impair the ability to address and understand mechanisms behind associations of social integration and neighborhood environment [27].

## Methods

### Aim

The aim of this study is first to describe the levels of social integration among middle-aged and older residents in a social housing area selected for urban regeneration. Second, to investigate whether social integration is associated with subjective evaluations of neighborhood dissatisfaction and unsafety. In the present study it is hypothesized that low levels of social integration are associated with an increased risk of neighborhood dissatisfaction and unsafety compared to high levels of social integration among middle-aged and older social housing residents.

### Study setting

The present study is conducted as part of a research project entitled, ‘Health, well-being and social relations in a changing neighborhood’ [28]. The social housing area was constructed between 1970 and 1973 and is located in the west end of Copenhagen, Denmark. It comprises 915 apartments, accommodating almost 2600 residents of various nationalities and many in disadvantaged socioeconomic positions [28]. A national policy directive from 2018 stipulated several social housing areas among these our study area to undergo urban regeneration [28]. This entails the relocation of residents and the demolition of housing with the aim of changing the resident composition and the built environment [28]. This particular neighborhood was selected as the study setting because it was one of the first of 15 social housing areas to initiate the urban regeneration program according to the national policy directive [28].

### Data material

#### Survey questionnaire

The survey questionnaire, ‘Health, well-being and social relations in a changing neighborhood’, was designed to collect self-reported information on demography, socioeconomic position, social relationships, health behavior and illness [27, 28]. The questionnaire employed validated instruments and items that have previously been applied in comparable study populations [27, 28]. The baseline information was collected between September 2018 and January 2019 prior to the urban regeneration program [27]. The questionnaire was translated from Danish to the seven most prominent language groups among the residents (Arabic, English, Pashto, Polish, Turkish, Urdu and Vietnamese) by two independent translators who then agreed on the final version [27].

The questionnaire was administered in-person by trained bilingual interviewers among all residents aged 45 years and above identified through the Civil Registration System [27]. The target group of residents aged 45 years and above was selected based on the findings from a qualitative study in the social housing area [29]. This study identified a need for research-based activities among middle-aged and older social housing residents [29]. Furthermore, it has been shown that social relations change throughout the course of life [6]. This highlights the importance of investigating the levels of social integration and the association between social integration and perceived neighborhood environment among middle-aged and older adults as a separate target group [6]. The interviewers went door-to-door at different hours during working days and weekends to comply with the needs of the residents until either the questionnaire was answered or participation declined [27]. The average attempt of contacts was 3.14 (interquartile range 1;4). Among the 604 invited residents a total of 209 answered the survey questionnaire (response rate: 35%) [27]. A subpopulation of 206 residents was selected for analyses of neighborhood dissatisfaction and unsafety, since three residents were excluded due to missing information on one or more variables.

### Variables

#### Social integration

**Fig. 1:**
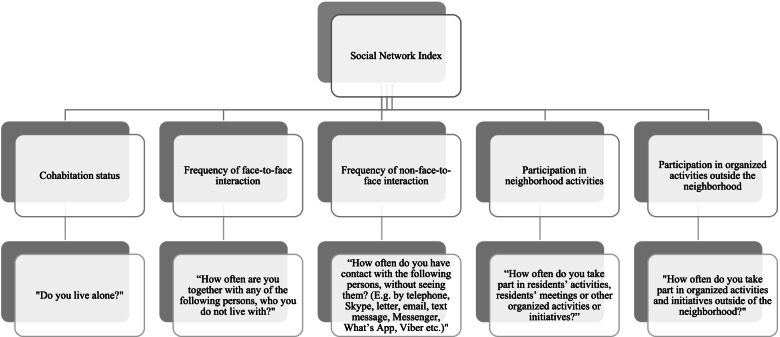
Social Network Index (Inspired by Berkman and Syme [7], the index was divided into five domains. For the questions “How often are you together with any of the following persons, who you do not live with?” and “How often do you have contact with the following persons, without seeing them? (E.g. by telephone, Skype, letter, email, text message, Messenger, What’s App, Viber etc.)” six items were listed, including partner or spouse, children or grandchildren, parents or parents-inlaw, other family members, e.g. uncle, aunt, cousin, brother-in-law, sister-inlaw, friends and neighbors, or other residents.)

The assessment of social integration was based on a quantitative measure [1]. A modified version of the Berkman and Syme Social Network Index (SNI) was used to assess the level of social integration [7]. The modified version of the SNI was constructed by five domains: “cohabitation status”, “frequency of face-to-face interaction”, “frequency of non-face-to-face interaction”, “participation in neighborhood activities” and “participation in organized activities outside the neighborhood” (Fig. 1: Social Network Index). The domains were assessed through five questions including 15 items. An additional file presents an overview of the questions, response categories and the point system used to assess the levels of social integration among the respondents (see Additional File 1).

According to the standard described by Berkman and Syme [7], our SNI considered not only the existence of social relations, but also their relative importance [9, 10]. Respondents that reported cohabitating were assigned four points to reflect the importance of this social tie related to the higher likelihood of access to interaction [7, 9]. The questions related to frequency of face-to-face and non-face-to-face interaction assessed six different social relations: 1) partner or spouse, 2) children or grandchildren, 3) parents or parents-in-law, 4) other family members (uncle, aunt, cousin, brother-in-law, and sister-in-law), 5) friends and 6) neighbors. If respondents reported face-to-face interaction with a social tie, that they did not live with, at least once a week, indicating high contact frequency, they were assigned two points for each type of social tie. If respondents reported non-face-to-face interaction with a social tie at least once a week, indicating high contact frequency, they were assigned two points for each type of social tie. The participation measures assessed frequency of participation in various activities. If respondents reported participation in neighborhood activities at least once a month, indicating high frequency of participation, they were assigned two points. The same applied for participation in organized activities outside the neighborhood. A composition score inspired by the Berkman and Syme SNI was calculated based on the number of points ranging from 0 to 30 points [7].

The relationship between SNI and the two outcomes, neighborhood dissatisfaction and unsafety, did not fit a linear (in continuous or deciles), quantile and cubic form. Therefore, SNI was included as a categorical variable with three categories based on the Berkman and Syme SNI [7]: low (0–10 points), medium (11–19 points), and high (20–30 points) as the reference group. An examination of the form of the relationships between each of four continuous SNI domains and the two outcomes, showed that face-to-face interaction as the only exposure variable could fit a linear relationship with both outcomes, respectively. However, when examining this relationship in detail by including face-to-face interaction as a categorical variable with six categories according to the point system, a clear pattern was only seen for the outcome, neighborhood dissatisfaction. As a consequence, all four exposure variables (face-to-face interaction, non-face-to-face interaction, participation in neighborhood activities, and participation in organized activities outside the neighborhood) were included as categorical variables with three categories: low, medium and high in the main analyses. An additional analysis with face-to-face interaction as a continuous variable is included.

#### Neighborhood dissatisfaction

Neighborhood dissatisfaction was assessed by the following question: “How satisfied, or dissatisfied are you with living in the neighborhood?” The response categories were: 1. Very dissatisfied; 2. Mainly dissatisfied; 3. Neither satisfied nor dissatisfied; 4. Mainly satisfied and 5. Very satisfied. Response categories were dichotomized into (0) Neighborhood dissatisfaction (1–3) and (1) Neighborhood satisfaction (4–5).

#### Neighborhood unsafety

Neighborhood unsafety was assessed by the following question: “To what extent do you feel safe when you are outdoors in the neighborhood?” The response categories were: 1. To a very low extent; 2. To a low extent; 3. To a certain extent; 4. To a high extent and 5. To a very high extent. Response categories were dichotomized into (0) Neighborhood unsafety (1–3) and (1) Neighborhood safety (4–5).

#### Sociodemographic characteristics

Demographic information included sex, age and country of birth. The latter was categorized into Western and non-Western origin according to the definition provided by Statistics Denmark [30]. Few respondents represented in each of the 44 countries of birth limited the possibility of other categorizations. The assessment of socioeconomic position was based on educational attainment and employment status. Educational attainment was measured by the highest level of education completed in Denmark or in another country, and was classified according to the UNESCO categories into the following three: ≤ 10 years of education, 11–15 years of education and > 15 years of education [31]. Employment status was assessed by asking the respondents about their current employment status and was dichotomized into employed and unemployed.

### Statistical analysis

Descriptive statistics including frequency measures were first conducted to illustrate the distribution of the exposure and outcome variables among the study population. Multiple logistic regression analyses of the association between social integration, each of five separate domains of SNI and neighborhood dissatisfaction and unsafety adjusted for age, sex, country of origin, educational attainment and employment status were conducted. The results from the multiple logistic regression analyses are presented as odds ratios (OR) with 95% confidence intervals (95% CI).

To test the robustness of the main results, four supplementary analyses were conducted. Firstly, the response category “medium” was selected as the reference group in preference to “high” for the social integration measure. Secondly, to test the robustness of our outcome measures, the response category “3. Neither satisfied nor dissatisfied” was moved from “(0) Neighborhood dissatisfaction” to “(1) Neighborhood satisfaction” and the response category “3.To a certain extent” was moved from “(0) Neighborhood unsafety” to “(1) Neighborhood safety”. Thirdly, educational attainment and employment status may act as both confounders and mediators. Therefore, three separate sensitivity analyses excluding first educational attainment and employment status, second educational attainment and third employment status from the adjusted logistic regression models were conducted. Finally, a non-response analysis was conducted to assess the distribution of demographic characteristics of the respondents and the full population regarding sex, age and country of origin.

## Results

### Descriptive results

The sociodemographic characteristics and perceptions of neighborhood dissatisfaction and unsafety among the study population are presented in Table 1. The mean age of respondents was 58.9 years, 67.5% were of non-Western origin, 63.6% had below 10 years of education and 66.5% were currently unemployed. A total of 36.4% reported being dissatisfied with living in the neighborhood and 31.1% reported feeling unsafe when outdoors in the neighborhood. The correlation between neighborhood dissatisfaction and unsafety is 0.31.

**Table 1:** Sociodemographic Characteristics and Perceptions of Neighborhood Dissatisfaction and Unsafety among the Study Population (*N* = 206)

**Sex**	**N (%)**
Male	102 (49.5)
Female	104 (50.5)
**Age**	**Mean (SD)**
Age in years (range 45; 88)	58.9 (10.5)
**Country of Origin**	**N (%)**
Western	67 (32.5)
Non-Western	139 (67.5)
**Education**	**N (%)**
≤ 10 years	131 (63.6)
11–15 years	45 (21.8)
> 15 years	30 (14.6)
**Employment Status**	**N (%)**
Employed	69 (33.5)
Unemployed	137 (66.5)
**Neighborhood Dissatisfaction**	**N (%)**
Satisfied	131 (63.6)
Dissatisfied	75 (36.4)
**Neighborhood Unsafety**	**N (%)**
Safe	142 (68.9)
Unsafe	64 (31.1)

The level of social integration and each of five SNI domains among the study population are presented in Table 2. A total of 23.8% of the respondents reported low levels of social integration. The mean SNI score among the respondents was 14.7 points (interquartile range 11; 19). A total of 26.7% reported living alone. Among the study population, 31.0% reported a low frequency of face-to-face interaction with family, friends and neighbors, whereas 17.5% reported a low frequency of non-face-to-face interaction. A total of 51.5% reported low a participation in neighborhood activities and 62.6% reported a low participation in organized activities outside the neighborhood.

**Table 2:** Characteristics of the Study Population Regarding Social Integration (*N* = 206)

**Social Integration**	**N (%)**
High	41 (19.9)
Medium	116 (56.3)
Low	49 (23.8)
**Social Network Index**	**Mean (SD)**
Interquartile range 11;19	14.7 (5.7)
**Cohabitation Status**	**N (%)**
Cohabitating	151 (73.3)
Living alone	55 (26.7)
**Frequency of Face-to-face Interaction**	**N (%)**
High	57 (27.7)
Medium	85 (41.3)
Low	64 (31.0)
**Frequency of Non-face-to-face Interaction**	**N (%)**
High	69 (33.5)
Medium	101 (49.0)
Low	36 (17.5)
**Participation in Neighborhood Activities**	**N (%)**
High	32 (15.5)
Medium	68 (33.0)
Low	106 (51.5)
**Participation in Organized Activities Outside the Neighborhood**	**N (%)**
High	27 (13.1)
Medium	50 (24.3)
Low	129 (62.6)

### Logistic regression results

The OR including 95% CI of the 12 separate associations between social integration, each of five domains of SNI and neighborhood dissatisfaction and unsafety, respectively, are presented in Table 3.

**Table 3:** Associations Between Social Integration, Five Separate Domains of SNI, Neighborhood Dissatisfaction and Unsafety^a^ (*N* = 206)

	Neighborhood Dissatisfaction				Neighborhood Unsafety			
	Unadjusted		Adjusted^b^		Unadjusted		Adjusted^b^	
	OR	(95% CI)	OR	(95% CI)	OR	(95% CI)	OR	(95% CI)
**Social Integration**								
High	1.00	(ref)	1.00	(ref)	1.00	(ref)	1.00	(ref)
Medium	**2.27**	**(1.02–5.06)**	**2.36**	**(1.04–5.38)**	1.33	(0.60–2.93)	1.51	(0.66–3.43)
Low	1.42	(0.56–3.58)	1.99	(0.75–5.34)	1.25	(0.50–3.10)	1.60	(0.59–4.33)
**Cohabitation Status**								
Cohabitating	1.00	(ref)	1	(ref)	1.00	(ref)	1.00	(ref)
Living alone	0.63	(0.32–1.23)	0.71	(0.33–1.52)	0.75	(0.38–1.48)	0.88	(0.40–1.94)
**Frequency of Face-to-face Interaction**^c^								
High	1.00	(ref)	1.00	(ref)	1.00	(ref)	1.00	(ref)
Medium	**2.26**	**(1.06–4.80)**	2.00	(0.91–4.38)	1.33	(0.61–2.09)	1.16	(0.50–2.53)
Low	**2.79**	**(1.26–6.18)**	**2.65**	**(1.16–6.06)**	**2.61**	**(1.78–5.79)**	**2.41**	**(1.04–5.57)**
**Frequency of Non-face-to-face Interaction**								
High	1.00	(ref)	1.00	(ref)	1.00	(ref)	1.00	(ref)
Medium	1.63	(0.85–3.11)	1.70	(0.87–3.31)	1.34	(0.68–2.61)	1.43	(0.71–2.88)
Low	1.14	(0.48–2.71)	1.32	(0.53–3.26)	1.16	(0.48–2.80)	1.34	(0.52–3.49)
**Participation in Neighborhood Activities**								
High	1.00	(ref)	1.00	(ref)	1.00	(ref)	1.00	(ref)
Medium	1.03	(0.42–2.54)	0.93	(0.36–2.38)	0.91	(0.34–2.40)	0.93	(0.34–2.59)
Low	1.48	(0.64–3.43)	1.46	(0.61–3.50)	1.86	(0.77–4.54)	1.81	(0.71–4.58)
**Participation in Organized Activities Outside the Neighborhood**								
High	1.00	(ref)	1.00	(ref)	1.00	(ref)	1.00	(ref)
Medium	1.41	(0.53–3.73)	1.45	(0.51–4.06)	1.20	(0.46–3.13)	1.28	(0.46–3.55)
Low	1.16	(0.48–2.76)	1.40	(0.54–3.63)	0.66	(0.28–1.58)	0.66	(0.25–1.73)

Values highlighted in bold indicate statistically significant estimates

*OR* Odds ratio, *95% CI* 95% Confidence Intervals

^a^Logistic regression results from 12 separate analyses of social integration and neighborhood dissatisfaction and unsafety, cohabitation and neighborhood dissatisfaction and unsafety, frequency of face-to-face interaction neighborhood dissatisfaction and unsafety, frequency of non-face-to-face interaction neighborhood dissatisfaction and unsafety, participation in neighborhood activities and neighborhood dissatisfaction and unsafety and participation in organized activities and initiatives outside the neighborhood and neighborhood dissatisfaction and unsafety

^b^ Adjusted for age, sex, country of origin, educational attainment and employment status

^c^Point estimates for the association between frequency of face-to-face interaction as a continuous variable and neighborhood dissatisfaction are OR: 0.78, 95% CI: 0.64–0.95

A medium level of social integration was associated with 2.36 (95% CI: 1.04–5.38) higher adjusted odds of neighborhood dissatisfaction compared to highest level of social integration. The point estimate of the association between low levels of social integration and neighborhood dissatisfaction was slightly lower and not statistically significant (OR: 1.99; 95% CI: 0.75–5.34). The point estimates of the associations between levels of social integration and neighborhood unsafety were imprecisely estimated and not statistically significant (low vs. high OR: 1.60; 95% CI: 0.59–4.33).

Living alone was associated with lower odds of neighborhood dissatisfaction and unsafety compared to cohabiting; however, estimates were imprecisely estimated and not statistically significant. A low frequency of face-to-face interaction was associated with 2.65 (95% CI: 1.16–6.06) higher odds of neighborhood dissatisfaction compared to high frequency of face-to-face interaction. The additional analysis of the association between frequency of face-to-face interaction as a continuous variable and neighborhood dissatisfaction provided an OR of 0.78 (95% CI: 0.64–0.95), meaning the higher level of social integration the lower level of dissatisfaction. A low frequency of face-to-face interaction was associated with 2.41 (95% CI: 1.04–5.57) higher odds of neighborhood unsafety compared to high frequency of face-to-face interaction. The point estimates of the association between low frequency of non-face-to-face interaction and neighborhood dissatisfaction and unsafety were lower and not statistically significant. There were no clear patterns regarding the point estimates for the association between participation in neighborhood activities and participation in organized activities outside the neighborhood, respectively, and the outcomes neighborhood dissatisfaction and unsafety.

### Supplementary analyses

The supplementary analysis in which the reference group for social integration was changed from “high” to “medium” is shown in Additional File 2. This analysis showed no notable differences in the associations between social integration and neighborhood dissatisfaction and unsafety. For the supplementary analysis predicting neighborhood dissatisfaction as a function of social integration, ORs were slightly higher for medium and low levels of social integration than those reported in Table 3, when changing the response category “3. Neither satisfied nor dissatisfied” from “(0) Neighborhood dissatisfaction” to “(1) Neighborhood satisfaction”. These estimates were nevertheless not statistically significant. For the supplementary analysis predicting neighborhood unsafety as a function of social integration, ORs were slightly increased for low levels of social integration than those reported in Table 3, when moving the response category “3. To a certain extent” from the outcome category “(0) Neighborhood unsafety” to “(1) Neighborhood safety”. However, these point estimates were not statistically significant. Additional File 3 provides the estimates of these supplementary analyses for the outcome measures. The supplementary analyses excluding first educational attainment and employment status, second educational attainment and third employment status from the adjusted logistic regression models showed results similar to those from the main models presented in Table 3. Data not shown. The demographic characteristics of the respondents and the full population are presented in Additional File 4. The analysis showed that the study sample of respondents was overall representative regarding the demographic characteristics of the full population in the social housing area, with the only difference in the distribution of sex. The male residents were slightly underrepresented among the respondents in the survey sample, while the female residents were overrepresented.

## Discussion

Based on a comprehensive interviewer-administrated survey questionnaire in multiple languages, we investigated the levels and importance of social integration for neighborhood dissatisfaction and unsafety among middle-aged and older adults in a social housing area in Denmark.

A growing body of public health literature investigates the relationship between specific characteristics of the social environment and neighborhood perceptions [8]. However, as emphasized by de Jesus and colleagues, very few empirical studies have examined the levels of social integration and the association between social integration and perceived neighborhood dissatisfaction and unsafety, particularly among residents in disadvantaged neighborhoods [8]. The present study contributes to the public health literature by demonstrating the levels of social integration among an underrepresented population group, residents in a social housing area. Furthermore, this study provides insights into the association between social integration—the five separate domains of SNI—and perceptions of neighborhood dissatisfaction and unsafety as a pathway through which social integration might influence health and well-being. Considering the importance of social integration for health and well-being [1–6], the results of this study could serve to develop targeted health promotion interventions that modify the frequency of face-to-face interaction [6]. Previous studies show that interventions promoting face-to-face interaction increase social connection and improve mental health [6].

### Social integration in disadvantaged neighborhoods

A total of 23.8% of respondents in our study reported low levels of social integration, which is considered high compared to the general population in Denmark [4]. However, the low levels of social integration identified in the present study is in line with previous studies conducted in disadvantaged neighborhoods in Denmark [2, 3]. Low levels of social integration have been found to be most prevalent among residents aged 65 years and above [3], and 40.8% of our study population consisted of residents aged 60 years and above. Older adults may be at increased risk of low social integration, since they often live alone and experience the loss of family members and friends [3].

### Social integration and neighborhood dissatisfaction

A total of 36.4% of respondents in our study was dissatisfied with living in the neighborhood. Similar levels of neighborhood dissatisfaction have previously been identified in disadvantaged neighborhoods [11–14]. Our results showed that medium levels of social integration were significantly associated with neighborhood dissatisfaction. This surprising result might be related to our construction of the SNI, in which cohabitation status weighted more than the other four domains. We found that cohabitating was associated with an increased risk of neighborhood dissatisfaction compared to living alone; however, estimates were imprecisely estimated and not statistically significant. This could potentially explain the identified association between medium levels of social integration and neighborhood dissatisfaction. In line with our hypothesis, we found that a low frequency of face-to-face interaction was associated with higher odds of neighborhood dissatisfaction compared to high frequency of face-to-face interaction even after adjustment for potential confounders. Our results are in line with previous studies emphasizing the importance of face-to-face interaction for neighborhood dissatisfaction [11–14].

Although point estimates of the association between frequency of participation in neighborhood activities and neighborhood dissatisfaction were imprecise with wide CI, a low frequency of participation was associated with higher odds of neighborhood dissatisfaction compared to high frequency of participation. Participation in local association activities might foster a sense of social cohesion and connectedness among residents in disadvantaged neighborhoods that lead to strong collective efficacy and decreased levels of neighborhood dissatisfaction [11, 12]. We previously found that residents with no prior experience of participation in neighborhood activities expressed neighborhood dissatisfaction compared to residents who regularly participated in neighborhood activities [15]. However, after engaging in a community-based health promotion intervention, residents expressed increased neighborhood satisfaction, as they felt more connected to their neighbors [15].

### Social integration and neighborhood unsafety

A total of 31.1% of respondents reported feeling unsafe being outdoors in the neighborhood. Contrary to this, other studies have found higher levels of neighborhood unsafety among residents in disadvantaged neighborhoods in Boston and Pennsylvania, respectively [8, 22]. A cross-sectional survey study from Boston, Massachusetts found opposite to our results that low levels of social integration were associated with reduced neighborhood unsafety among male residents [8]. The authors explain that it is possible that the association between social integration and neighborhood unsafety does not follow a linear dose–response curve [8], which we confirmed in our analysis. Another possible explanation is that social integration can be characterized by negative social norms, influences and interpersonal interactions in disadvantaged neighborhoods, which contributes to neighborhood unsafety [8]. However, we did not investigate this explanation in our study.

We found that a low frequency of face-to-face interaction was associated with higher odds of neighborhood unsafety compared to high frequency of face-to-face interaction even after adjustment for important potential confounders. This indicates that spending time together with family, friends and neighbors has a positive impact on neighborhood safety leading to potential health benefits [25]. Although point estimates of the association between cohabitation status and neighborhood safety were imprecise with wide CI, living alone was associated with lower odds of neighborhood unsafety compared to cohabitating. Cohabitating with a spouse or partner and especially children might cause concern and worry that in turn may increase perceptions of neighborhood unsafety [8].

### Consequences of urban regeneration for social integration

Previous research has identified that high turnover of residents due to urban regeneration in disadvantaged neighborhoods might decrease place attachment, reduce social integration and erode feelings of trust and safety [16, 17, 20, 25, 32, 33]. Relocated residents experience several challenges to social integration, in which they struggle to establish new social relations and face stigmatization [16, 17]. For remaining residents, we previously identified reduced place attachment caused by urban regeneration [15]. The present study shows that a low frequency of face-to-face interaction is associated with neighborhood dissatisfaction and unsafety. Considering the prospective urban regeneration in the social housing area, loss of social relations among residents might increase neighborhood dissatisfaction and unsafety, which can negatively affect the health and well-being of residents [8].

### Methodological considerations

The results of this study should be interpreted based on the following considerations. First, the response rate was relatively low (34.1%) due to difficulties in reaching residents in the social housing area. The majority (67.5%) of the study population was of non-Western origin, and this corresponds well with the overall distribution of country of origin in the social housing area (Additional File 4). Previous research on social integration in disadvantaged neighborhoods in Denmark obtained a response rate between 15.1% – 25.7% among residents of non-Western origin [2, 3]. The relatively high response rate among residents of non-Western origin might be the fruits of our efforts on translating the survey into the seven most prominent languages and the use of trained bilingual interviewers [27]. The identified overrepresentation of female residents in the survey sample mirrors a typical trend in health surveys, because men have been found to be less willing to participate in such surveys compared to women [34].

Second, we cannot draw causal conclusions due to the cross-sectional and observational nature of this study. It is possible that respondents, who are already satisfied with and safe in the neighborhood are more socially integrated. Moreover, neighborhood satisfaction and safety may breed into social integration if respondents, who are satisfied and safe, have a higher frequency of face-to-face, non-face-to-face interaction and greater participation in various activities. To strengthening the results, these bi-directional relationships should be investigated with longitudinal data that follows respondents over several time points. Perceptions of neighborhood environment including neighborhood satisfaction and safety can be a potential pathway through which social integration affects health. Future studies with a longitudinal design among larger populations can investigate such pathways.

Thirdly, the narrow selection of measures of social integration might be a limitation. The single focus on quantity of relations is less likely to capture the complex nature of social relations among residents [1, 9, 10]. To further strengthen the results, the quality of social relations such as perceived support and loneliness needs to be included [1, 9, 10]. Research has demonstrated that social relations can be fraught with conflict and strain and thus harmful for health and well-being [1, 9, 10]. The present application of social integration is insensitive to conflict, since it does not explicitly examine the quality of social relations. Future research should therefore include quality indicators of perceived support and conflictual relations [1, 9, 10].

Despite these limitations, our study highlights the importance of frequency of face-to-face interaction for neighborhood dissatisfaction and unsafety among social housing residents. The self-reported data on social integration and neighborhood dissatisfaction and unsafety are essential to understand these associations, since all aspects are characterized by individual experiences. The specific instruments and items used in our questionnaire have been applied for several years in Danish health surveys [2–4, 27]. Neighborhood dissatisfaction and unsafety were assessed by single item questions, which has been recommended to capture perceptions of neighborhood environment among residents in disadvantaged neighborhoods [8, 35]. The correlation between neighborhood dissatisfaction and unsafety is relatively modest at 0.31, illustrating that the concepts are moderately related, but also measure different aspects of neighborhood environment.

### Implications for policy, practice and research in public health

It is important to maintain a policy focus on ensuring opportunities for social integration across populations, including residents in disadvantaged neighborhoods. Our study indicates the need for interventions to foster face-to-face interaction to reduce neighborhood dissatisfaction and unsafety and associated health consequences.

In terms of practice development, more focus on engaging residents in health promotion interventions, is needed. This is particularly important under the circumstances of urban regeneration, in which residents might feel unheard and neglected [16, 17]. Fostering social relations and local participation might be of particular importance, when the residents are subjected to urban regeneration [16, 17].

This study is based on cross-sectional survey data. Qualitative research is needed to obtain nuanced perspectives on social integration and neighborhood environment through long-term presence in disadvantaged neighborhoods. This will enable a better understanding of influencing pathways and identification of residents in need of health promotion interventions to reduce inequities in social integration and health.

## Conclusions

This study described the levels of social integration and the association between social integration and neighborhood dissatisfaction and unsafety among middle-aged and older social housing residents with different nationalities in disadvantaged socioeconomic positions. Almost one-fourth of respondents reported low levels of social integration. In accordance with our hypothesis, a medium level of social integration was associated with increased neighborhood dissatisfaction. A low frequency of face-to-face interaction was associated with increased neighborhood dissatisfaction and unsafety. A focus on health promotion approaches that create opportunities for neighbors to interact and build social relations is thus needed. Such health promotion interventions may enhance social relations and improve perceptions of neighborhood environment and quality of life among residents in disadvantaged neighborhoods.

## Supplementary Information

**Additional file 1:** **Table S1.** Construction of Social Network Index Inspired by Berkman and Syme.

**Additional file 2:** **Table S2.** Associations Between Social Integration and Neighborhood Dissatisfaction and Unsafety.

**Additional file 3:** **Table S3.** Associations Between Social Integration and Neighborhood Dissatisfaction and Unsafety.

**Additional file 4:** **Table S4. **Demographic Characteristics of Respondents and Full Population.

## Data Availability

The datasets generated and analyzed during the present study are not publicly available due to the Danish Data Protection Act, but are available from the last author (principal investigator) on reasonable request.

## Abbreviations

SNI: Social Network Index
OR: Odds Ratio
CI: Confidence Intervals
