# Supplementary material for: The association between social integration and neighborhood dissatisfaction and unsafety: a cross-sectional survey study among social housing residents in Denmark
Source: Arch Public Health. 2022 Aug 12;80:190. doi: 10.1186/s13690-022-00945-9 (PMC9373542; doi:10.1186/s13690-022-00945-9)
Supplement: Supplementary file 1 — Additional file 1: Table S1. Construction of Social Network Index Inspired by Berkman and Syme. [file 13690_2022_945_MOESM1_ESM.docx]

| **Table S1** Construction of Social Network Index Inspired by Berkman and Syme (1) | | | | |
| --- | --- | --- | --- | --- |
| **Domain** | **Questions and Items in Survey Questionnaire** | **Response Categories** | **Revised Response Categories** | **Points** |
| **Cohabitation Status** | “Do you live alone?” | 1. No  2. Yes | 1. No  2. Yes | 4: Cohabitating  0: Living alone |
| **Frequency of Face-to-face Interaction**  **Frequency of Non-face-to-face Interaction** | “How often are you together with any of the following persons, who you do not live with?”  a) Partner or spouse  b) Children or grandchildren  c) Parents or parents-in-law  d) Other family members, e.g. uncle, aunt, cousin, brother-in-law, sister-in-law  e) Friends  f) Neighbors, or other residents | 1. Several days a week  2. About once a week  3. One to three times a month  4. Less than once a month  5. Never  6. Not relevant | 1. Several days week/ About once a week  2. One to three times a month/ Less than once a month  3. Never/ Not relevant | 2: High contact frequency  1: Medium contact frequency  0: Low contact frequency |
|  | “How often do you have contact with the following persons, without seeing them? (E.g. by telephone, Skype, letter, email, text message, Messenger, What’s App, Viber etc.)”  a) Partner or spouse  b) Children or grandchildren  c) Parents or parents-in-law  d) Other family members, e.g. uncle, aunt, cousin, brother-in-law, sister-in-law  e) Friends  f) Neighbors, or other residents | 1. Several days a week  2. About once a week  3. One to three times a month  4. Less than once a month  5. Never  6. Not relevant | 1. Several days week/ About once a week  2. One to three times a month/ Less than once a month  3. Never/ Not relevant | 2: High contact frequency  1: Medium contact frequency  0: Low contact frequency |
| **Participation in Neighborhood Activities** | “How often do you take part in residents’ activities, residents’ meetings or other organized activities or initiatives?” | 1. Once a week or more  2. Between one and three times a month  3. Several times a year  4. Approximately once a year 5. Never | 1. Once a week or more/ Between one and three times a month  2. Several times a year/ Approximately once a year  3. Never | 2: High participation  1: Medium participation  0: Low participation |
| **Participation in Organized Activities Outside the Neighborhood** | “How often do you take part in organized activities and initiatives outside of the neighborhood?” | 1. Once a week or more  2. Between one and three times a month  3. Several times a year  4. Approximately once a year 5. Never | 1. Once a week or more/ Between one and three times a month  2. Several times a year/ Approximately once a year  3. Never | 2: High participation  1: Medium participation  0: Low participation |

1. Berkman L, Syme LS. Social networks, host resistance, and mortality: A nine-year follow-up study of alameda county residents. American Journal of Epidemiology. 1979;109(2):186–204.
