# Supplementary material for: The association between social integration and neighborhood dissatisfaction and unsafety: a cross-sectional survey study among social housing residents in Denmark
Source: Arch Public Health. 2022 Aug 12;80:190. doi: 10.1186/s13690-022-00945-9 (PMC9373542; doi:10.1186/s13690-022-00945-9)
Supplement: Supplementary file 2 — Additional file 2: Table S2. Associations Between Social Integration and Neighborhood Dissatisfaction and Unsafety. [file 13690_2022_945_MOESM2_ESM.docx]

| **Table S2** Associations Between Social Integration^1^ and Neighborhood Dissatisfaction and Unsafety (N=206) | | | | | | | | | | | |
| --- | --- | --- | --- | --- | --- | --- | --- | --- | --- | --- | --- |
|  | Neighborhood Dissatisfaction | | | | |  | Neighborhood Unsafety | | | | |
|  | Unadjusted | |  | Adjusted^a^ | |  | Unadjusted | |  | Adjusted^a^ | |
|  | OR | (95 % CI) |  | OR | (95 % CI) |  | OR | (95 % CI) |  | OR | (95 % CI) |
| **Social Integration** | | | | | | | | | | | |
| Medium | 1.00 | (ref) |  | 1.00 | (ref) |  | 1.00 | (ref) |  | 1.00 | (ref) |
| High | **0.44** | **(0.20-0.98)** |  | 0.424 | (0.19-0.97) |  | 0.753 | (0.34-1.66) |  | 0.662 | (0.29-1.51) |
| Low | 0.63 | (0.31-1.26) |  | 0.845 | (0.40-1.78) |  | 0.938 | (0.46-1.90) |  | 1.059 | (0.48-2.32) |

^1^Medium level of social integration as reference group
Values highlighted in bold indicate statistically significant estimates
OR: Odds ratio; 95% CI: 95% Confidence Intervals
^a^Adjusted for age, sex, country of origin, educational attainment and employment status
