# Supplementary material for: The association between social integration and neighborhood dissatisfaction and unsafety: a cross-sectional survey study among social housing residents in Denmark
Source: Arch Public Health. 2022 Aug 12;80:190. doi: 10.1186/s13690-022-00945-9 (PMC9373542; doi:10.1186/s13690-022-00945-9)
Supplement: Supplementary file 3 — Additional file 3: Table S3. Associations Between Social Integration and Neighborhood Dissatisfaction and Unsafety. [file 13690_2022_945_MOESM3_ESM.docx]

| **Table S3** Associations Between Social Integration and Neighborhood Dissatisfaction^1^ and Unsafety^2^ (N=206) | | | | | | | | | | | |
| --- | --- | --- | --- | --- | --- | --- | --- | --- | --- | --- | --- |
|  | Neighborhood Dissatisfaction | | | | |  | Neighborhood Unsafety | | | | |
|  | Unadjusted | |  | Adjusted^a^ | |  | Unadjusted | |  | Adjusted^a^ | |
|  | OR | (95 % CI) |  | OR | (95 % CI) |  | OR | (95 % CI) |  | OR | (95 % CI) |
| **Social Integration** | | | | | | | | | | | |
| High | 1.00 | (ref) |  | 1.00 | (ref) |  | 1.00 | (ref) |  | 1.00 | (ref) |
| Medium | 2.41 | (0.78-7.43) |  | 2.55 | (0.81-8.01) |  | 1.24 | (0.43-3.60) |  | 1.42 | (0.48-4.23) |
| Low | 1.72 | (0.48-6.18) |  | 2.31 | (0.61-8.70) |  | 1.54 | (0.47-5.02) |  | 1.94 | (0.55-6.88) |

^1^ For the outcome measure “Neighborhood Dissatisfaction,” the response category “3. Neither satisfied nor dissatisfied” is moved from “(0) Neighborhood dissatisfaction” to “(1) Neighborhood satisfaction.”

^2^For the outcome measure “Neighborhood Unsafety,” the response category “3.To a certain extent” is moved from “(0) Neighborhood unsafety” to “(1) Neighborhood safety.”
OR: Odds ratio; 95% CI: 95% Confidence Intervals
^a^ Adjusted for age, sex, country of origin, educational attainment and employment status
