# Supplementary material for: The association between social integration and neighborhood dissatisfaction and unsafety: a cross-sectional survey study among social housing residents in Denmark
Source: Arch Public Health. 2022 Aug 12;80:190. doi: 10.1186/s13690-022-00945-9 (PMC9373542; doi:10.1186/s13690-022-00945-9)
Supplement: Supplementary file 4 — Additional file 4: Table S4. Demographic Characteristics of Respondents and Full Population. [file 13690_2022_945_MOESM4_ESM.docx]

| **Table S4** Demographic Characteristics of Respondents and Full Population | | |
| --- | --- | --- |
|  | **N (%)** | |
|  | **Respondents** | **Full population** |
| **Total** | 206 (100) | 604 (100) |
| **Sex** |  |  |
| Male | 102 (49.5) | 322 (53.3) |
| Female | 104 (50.5) | 282 (46.7) |
| **Country of Origin** | | |
| Western | 67 (32.5) | 201 (33.3) |
| Non-Western | 139 (67.5) | 403 (66.7) |
|  | **Mean (SD)** | |
| **Age** |  |  |
| Age in years | 58.9 (10.5) | 58.0 (10.2) |
